# P-333. Assessing HIV Pre-Exposure Prophylaxis Coverage at a Midwest Federally Qualified Health Center (FQHC)

**DOI:** 10.1093/ofid/ofaf695.552

**Published:** 2026-01-11

**Authors:** Jessica Witt, Josh Havens, Sara H Bares, Jessica Downes, Jennifer M Davis, Alex T Dworak, Mark Carter, Shawnalyn Sunagawa

**Affiliations:** University of Nebraska Medical Center College of Pharmacy, Omaha, Nebraska; University of Nebraska Medical Center, Omaha, NE; University of Nebraska Medical Center, Omaha, NE; University of Nebraska Medical Center College of Pharmacy, Omaha, Nebraska; University of Nebraska Medical Center, Omaha, NE; OneWorld Community Health Centers, UNMC, Omaha, Nebraska; University of Nebraska Medical Center, Omaha, NE; University of Nebraska Medical Center, Omaha, NE

## Abstract

**Background:**

In the United States, Hispanic/Latine persons are disproportionately affected by new HIV infections, underscoring the need for targeted HIV pre-exposure prophylaxis (PrEP) implementation to optimize uptake in the most at-risk populations. OneWorld (OW), a FQHC, primarily serves a Hispanic/Latine population and currently offers PrEP services. This study evaluated OW’s current PrEP utilization in comparison to patients receiving care at OW who are at risk for HIV acquisition.

**Figure:**
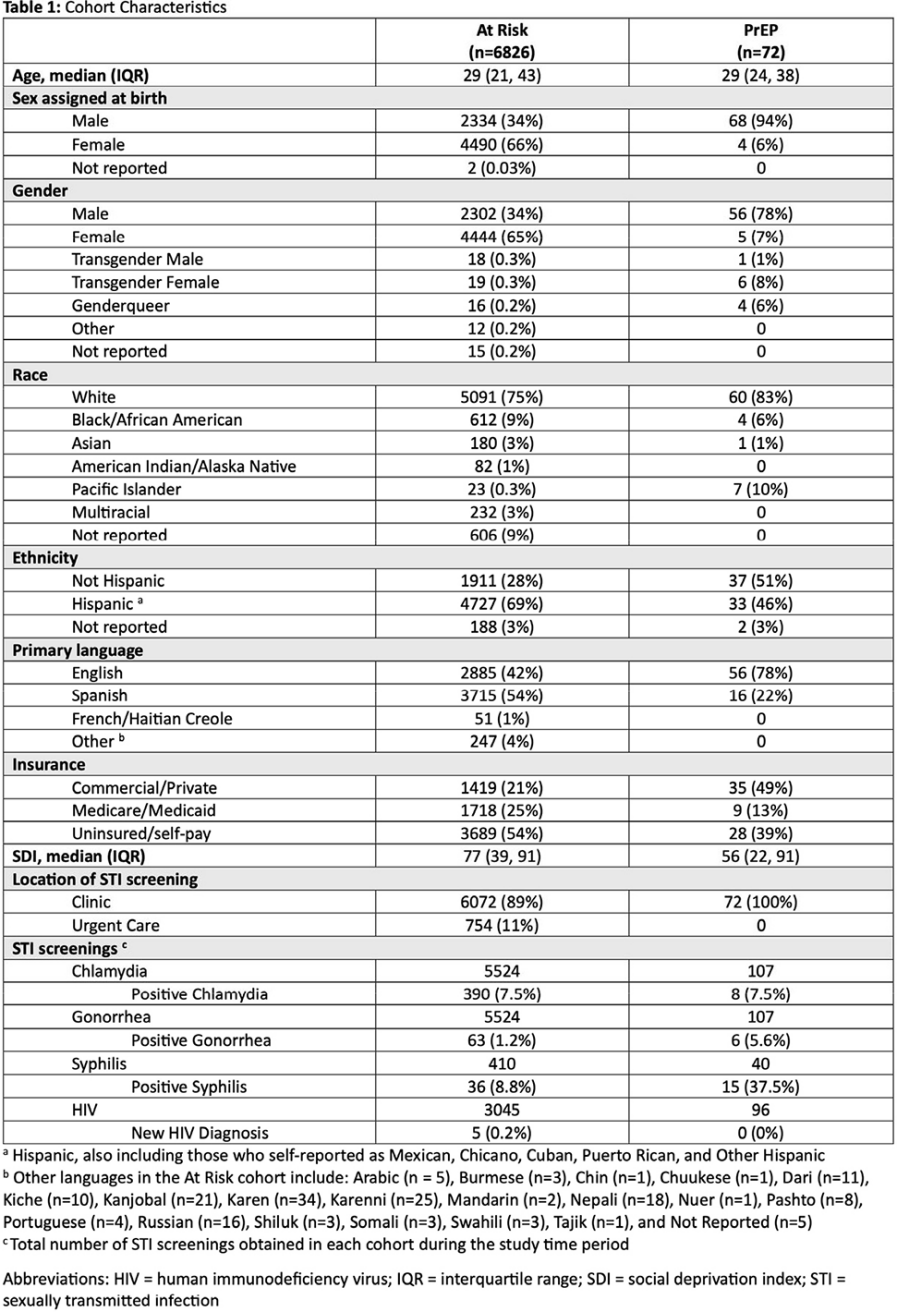

**Figure:**
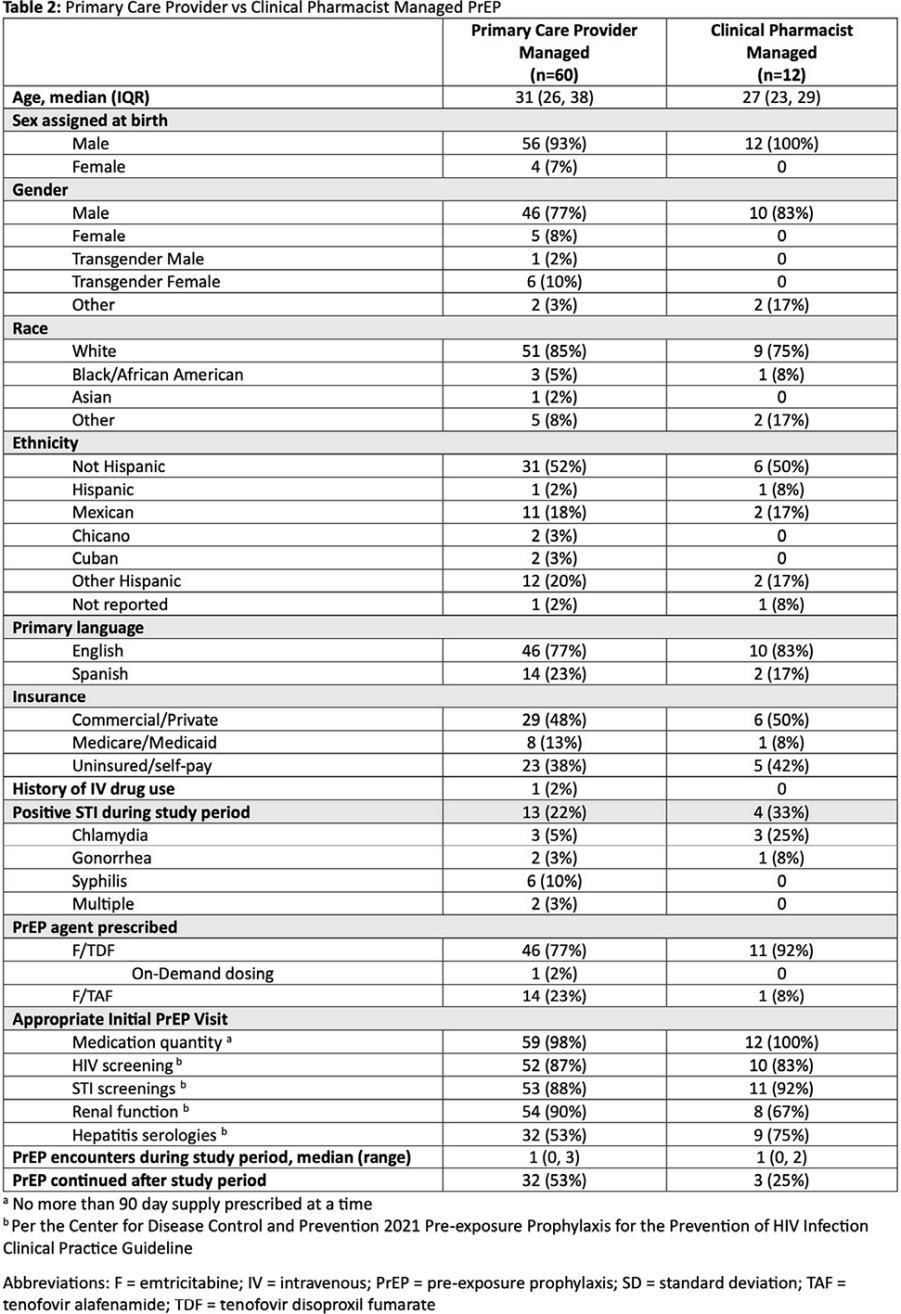

**Methods:**

We conducted a retrospective evaluation of patients at risk of acquiring HIV between January 1 and June 30, 2024. The at-risk population was defined as any patient receiving PrEP or non-occupational post-exposure prophylaxis services, those with sexually transmitted infection (STI) screenings or diagnoses, injection drug use, or a new HIV diagnosis. Pregnant patients were excluded since they are currently ineligible for PrEP services at OW. The primary outcome was PrEP coverage, defined as proportion of patients receiving PrEP services compared to patients at risk of acquiring HIV. Secondary outcomes included proportion of patients started on PrEP by a primary care provider and referred for clinical pharmacist co-management, rates of appropriate guideline-directed screenings at initial visit, and PrEP continuation post-study period. Descriptive statistics summarized cohort characteristics and outcomes.

**Results:**

Cohort characteristics are listed in Table 1. Only 1% (n=72/6826) of patients at risk of acquiring HIV were prescribed PrEP. Non-Hispanic patients accounted for 51% of PrEP recipients despite comprising only 28% of the at-risk population. Similarly, females accounted for 65% of those at risk, but made up only 7% of those prescribed PrEP. A small proportion (17%) of patients prescribed PrEP were referred for clinical pharmacist co-management. Proportion of appropriate guideline directed initial screenings are listed in Table 2. Only 49% (n=35/72) of patients continued PrEP at OW beyond the study period.

**Conclusion:**

There is a need for increased PrEP access and prescribing at OW, particularly among Hispanic/Latine and female patients who represent a majority of the at-risk population but less than half of PrEP recipients. This highlights an area for targeted intervention.

**Disclosures:**

Josh Havens, PharmD, Gilead Sciences: Grant/Research Support|Merck: Advisor/Consultant|ViiV Healthcare: Advisor/Consultant Jennifer M. Davis, MD, Viiv: Grant/Research Support

